# Influence of the surrounding medium on the luminescence-based thermometric properties of single Yb^3+^/Er^3+^ codoped yttria nanocrystals†

**DOI:** 10.1039/d1na00466b

**Published:** 2021-09-16

**Authors:** Jefferson Augusto Oliveira Galindo, Allison Rodrigo Pessoa, Anderson Monteiro Amaral, Leonardo de Souza Menezes

**Affiliations:** Department of Physics, Universidade Federal de Pernambuco – UFPE 50670-901 Recife PE Brazil anderson.amaral@ufpe.br +55-81-2126-7640

## Abstract

While temperature measurements with nanometric spatial resolution can provide valuable information in several fields, most of the current literature using rare-earth based nanothermometers report ensemble-averaged data. Neglecting individual characteristics of each nanocrystal (NC) may lead to important inaccuracies in the temperature measurements. In this work, individual Yb^3+^/Er^3+^ codoped yttria NCs are characterized as nanothermometers when embedded in different environments (air, water and ethylene glycol) using the same 5 NCs in all measurements, applying the luminescence intensity ratio technique. The obtained results show that the nanothermometric behavior of each NC in water is equivalent to that in air, up to an overall brightness reduction related to a decrease in collected light. Also, it was observed that the thermometric parameters from each NC can be much more precisely determined than those from the “ensemble” equivalent to the set of 5 single NCs. The “ensemble” parameters have increased uncertainties mainly due to NC size-related variations, which we associate to differences in the surface/volume ratio. Besides the reduced parameter uncertainty, it was also noticed that the single-NC thermometric parameters are directly correlated to the NC brightness, with a dependence that is consistent with the expected variation in the surface/volume ratio. The relevance of surface effects also became evident when the NCs were embedded in ethylene glycol, for which a molecular vibrational mode can resonantly interact with the Er^3+^ ions electronic excited states used in the present experiments. The methods discussed herein are suitable for contactless on-site calibration of the NCs thermometric response. Therefore, this work can also be useful in the development of measurement and calibration protocols for several lanthanide-based nanothermometric systems.

## Abstract 2

5 individual Yb^3+^/Er^3+^ codoped yttria nanocrystals were characterized as luminescent nanothermometers when embedded in 3 environments (air, water, and ethylene glycol). The methods discussed are suitable for contactless on-site calibration.

## Graphical abstract

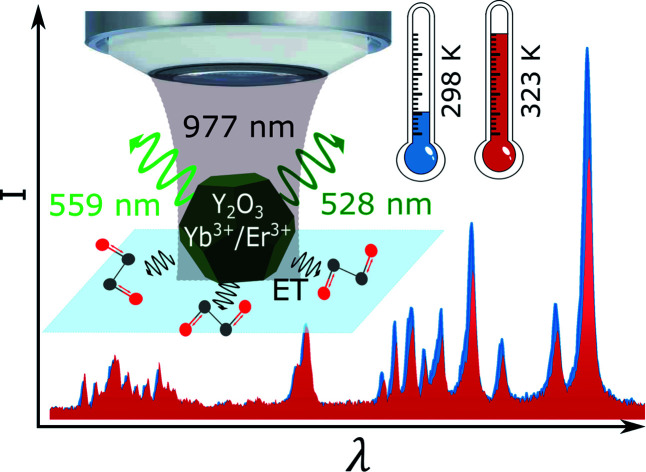

## 1 Introduction

Contactless nanoscale temperature sensors with both high spatial and thermal resolution have numerous applications.^1^ For example, they can be applied in microelectronics^2^ to perform micro/nano-electronic failure diagnosis; in microfluidics^3^ to create low-cost fast processing sensors^4^ and to detect instantaneous velocity of Brownian nanocrystals (NCs);^5^ in cell biology to perform *in vivo* temperature sensing of a single cell^6^ and diagnosis of tissue inflammation;^7^ to investigate methanol-to-hydrocarbons catalytic reactions,^8,9^ and beyond.^1,10–12^ Among current techniques for nanoscale temperature sensing, one of the most promising involves the frequency upconversion (UC) from lanthanide ions in the trivalent-state.^13–16^ UC is a nonlinear optical effect in which the ion emits photons with energies higher than those used for excitation^17^ and can be used for sensitive nanothermometers operating over a broad range of temperatures.^15,16^ While the codoped system Yb^3+^/Er^3+^ can be excited both with near-infrared (NIR) or ultraviolet radiation, the NIR-to-visible UC process is of widespread interest because the excitation can be performed using low cost light sources and in the biological transparency windows.^18^ Even though UC is a promising approach to nanothermometry, the vast majority of the achievements on the field in the past decades were obtained with particle ensembles,^10^ either in colloidal suspensions^19^ or bulk materials.^20,21^ While these studies give a broad perspective of the topic and indicate several potential applications, they do not necessarily can be applied directly to single NC thermometers. The behavior of individual NCs can change due to differences in size,^22,23^ surface effects,^23,24^ or doping concentration fluctuations,^25^ for example. Another important distinction may arise from the required excitation power per particle for single-particle sensing, which is often greater than those used for studies with colloidal suspensions or bulk materials. Therefore, practical single-NC thermometers may exhibit a distinct photophysics from those observed in bulk or NC ensembles, and this must be considered in applications.^10^ There are already some important achievements in the individual NC regime as in real-time temperature sensing in living cells,^6,26^ as an orientation sensor at a individual NC level^27^ and as super-resolution nanoscopic temperature sensors.^28,29^

Particularly, for luminescence intensity ratio (LIR) measurements, ensembles of NCs lead to outstanding sensitivity values (up to^2^ 9.6% K^−1^) and can be directly applied in problems as *in vivo* temperature measurements.^30–32^ However, even though the 4f–4f transitions in rare-earth ions are somewhat shielded from the environment^23^ and the LIR is a ratiometric measurement technique, these features are insufficient to correctly calibrate individual NCs as thermometers. For instance, previous LIR temperature measurements in lanthanide complexes show that the NC size affects the sensitivity of the nanothermometers.^33^ As another example, Zhou and collaborators detected different spectroscopic signatures for individual NCs and ensembles^28^ which they associated to the nonlinear nature of UC systems. Therefore, these factors indicate that even adjacent NCs may have different thermal responses and each must be calibrated/characterized individually. Otherwise, it becomes likely to obtain temperature misreadings even when measuring thermal features in the micrometer scale.^10^ In this sense, the single-particle approach can avoid these issues because it is a well-characterized and calibrated nanometric sensor that can be used as an universal probe.^6,26^

The NC environment also plays an important role when working with luminescent sensors as nano-probes in biological and complex chemical media. The behavior of luminescent centers close to the surface may change significantly due to NC surface defects^23^ or quenching induced by energy transfer to vibrational modes of nearby molecules.^24^ For instance, *in vivo* measurements can show biased or inaccurate sensing due to its intricate chemical composition.^11^ In another study, Stripka *et al.*^34^ measured concentration-dependent sensitivities for lanthanide nanothermometers suspended in a solution containing water and heavy water (D_2_O). The authors proposed that the distinct sensitivities can be explained by the different energy mismatches between H–O and D–O bonds with respect to a radiative transition. Therefore, the investigation of the emission properties in different media must be performed to adequately understand the NC-environment interactions in individual NC sensing systems.

The properties of the Ln^3+^ ions' matrix is also important for the thermometer characteristics. Crystalline lattices with low-energy phonon modes reduce the non-radiative multiphonon relaxation rates and consequently increases the overall luminescence detection.^35^ Yttria (Y_2_O_3_) presents these characteristics^36^ and is widely studied due to its relatively high physical and chemical stability,^37^ low toxicity,^38,39^ biocompatibility^40,41^ and additionally offers a high solubility for Ln^3+^ ions,^36^ which makes this host matrix excellent for biological applications.

The present work reports results on five different single NCs of codoped yttria [Y_2_O_3_:Yb^3+^ (1.5%)Er^3+^ (0.5%)] used as luminescent nanothermometers through the LIR technique. The NCs were prepared as discussed in the Methods. To determine the LIR, the NCs were excited by a home-assembled CW fiber laser emitting at 977 nm. In the investigated NCs the Yb^3+^ ions act as sensitizers, efficiently absorbing the NIR radiation and transferring to the Er^3+^ ions, which acts as luminescent emitters in the red and green spectral bands.^26,35^ The LIR was determined using transitions in the green, from which the relative thermal sensitivity and the thermal resolution for each NC were characterized. It was also determined how each nanothermometer behaves when embedded in three distinct environments (air, water and ethylene glycol). To be sure that the same five NCs were used, their relative positions were identified with the use of a home-made sample-scanning inverted optical microscope. The scanning luminescence images confirm that the investigated NCs constellation remained identical in the three environments, thus indicating that the experiments were performed with the same NCs. The relative sensitivities in air and water reported in this work are among the highest measured values for this kind of system.^14,25^ While the thermal response for each NC in water and air are observed to be quite similar, some differences appear when the NCs are embedded in ethylene glycol. This behavior can be explained through the energy transfer from the Er^3+^ excited states to the molecular C–O bond stretching vibrational modes of ethylene glycol molecules adsorbed to the yttria NCs, as discussed below. It was also noticed that the thermal response varies with the total NC brightness, which can be associated with the slight variations in the surface/volume ratio among the NCs with different sizes. This is particularly important since it allows to calibrate one particle in an ensemble and adjust the thermal response of the remaining particles using only the total particle luminescence measurements. These results and some specific features of individual particles are discussed in terms of the potential applicability of such NCs for thermal sensing.

## 2 Methods

### 2.1 Sample preparation

The single NCs studied in this work were from the same batch as those investigated in the ref. 36, in which the NCs synthesis procedure is described in detail. SEM and TEM images of the NCs can also be found, as well as Raman spectroscopy results for codoped and pristine Y_2_O_3_ NCs synthesized for this study (see Fig. S1 on ESI† and ref. 36). The NCs have a spherical shape and body-centered cubic crystalline structure, in addition they also present high phase purity and homogeneous distribution of the rare earth ions. Here, the samples were prepared by suspending 0.01 g of a Yb^3+^/Er^3+^:Y_2_O_3_ NCs powder in 1 mL of isopropyl alcohol. The as-prepared NCs are monodisperse and have an average size of 120 nm ± 20 nm. The size distribution of the NCs resulting from this synthesis protocol is presented in the Fig. S1 on ESI.† The dispersion is then ultrasonicated for 15 minutes to avoid the formation of agglomerates. After sonication, the dispersion is stored during 24 h for decantation. Borosilicate glass coverslips (Menzel-Gläser *#*1) were cleaned with a special cleaning solution (Hellmanex III) and once the decantation time is reached, 10 μL of the dispersion is spin-coated on a coverslip in two steps, for 6 s at 2000 rpm and then 15 s at 3200 rpm. As can be seen in the ref. 36, this deposition procedure leads to a good distribution of individual NCs, 2–4 μm apart from each other.

### 2.2 Experimental setup

**Fig. 1:**
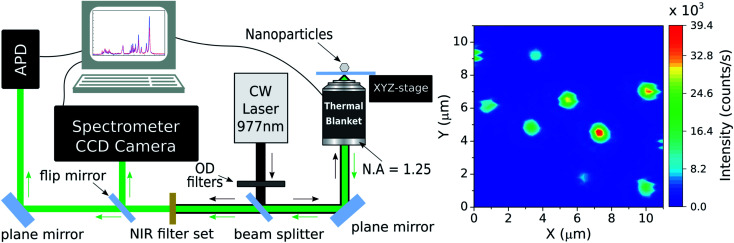
(Left) experimental apparatus scheme representing the inverted sample-scanning optical microscope used in this work. OD: optical neutral density filters; APD: avalanche photodiode; N.A.: numerical aperture. (Right) scanning luminescence image of codoped Yb^3+^/Er^3+^ NCs obtained with the inverted sample-scanning microscope setup. The figure shows a 11 × 11 μm region with 60 pixels in *x* and *y* directions and an integration time of 30 ms per pixel. The colorbar indicates the time averaged intensity. The white arrows designate the selected NCs with which the nanothermometry experiments were made. The pump power density used here was 6 × 10^3^ W cm^−2^ at the sample.

In order to work with individual NCs a luminescence microscopy setup was assembled. Such apparatus can be seen in the Fig. 1 and it is similar to that used in.^42^ In the present work, the excitation laser beam (home-assembled CW fiber laser emitting at 977 nm) passes through a set of neutral density absorptive filters for power control and is sent to the home-made inverted optical microscope by a 50 : 50 beam splitter (Thorlabs BS1). The excitation light is tightly focused on the coverslip containing the NCs by a high numerical aperture microscope objective (Edmund Optics 43905 100× N.A. = 1.25/oil immersion). The spatial position of the sample is determined by a computer-controlled piezoelectric 3D stage (Piezosystem Jena TRITOR 100 T-403-00) which allows the sample to be raster scanned. The same objective lens is used to collect the fluorescence of the detected NCs and this signal, along with the laser light reflected by the glass coverslip, is sent backward to the detection system. After the collected light crosses the beam splitter the luminescence is filtered by a set of NIR shortpass optical filters (Thorlabs FES0750 and Semrock FF01-842-sp-25).

The NCs luminescence can then be sent to two different detection paths. The first one directs the light to an avalanche photodiode (APD, idQuantique 100-50) which is used to perform single photon detection and luminescence intensity measurements on individual NCs. Those emission measurements associated with the position control of the sample enable the acquisition of the two-dimensional, scanning luminescence images of the NCs. The second path sends the light emitted by the single NCs to a spectrometer (Princeton Instruments SP2500) coupled to a CCD camera (Andor DU401-BV). By selecting this detection path one can perform the spectral analysis of fluorescence emitted by the NCs. Finally, the signal from both detection systems are collected by a computer for data analysis.

### 2.3 Experimental methods

The temperature of the sample is controlled by a home-made heating device that consists of a thermal blanket that embraces the microscope objective. The device is computer-controlled and the local heating of the sample is indirect, happening *via* the immersion oil and thus being necessary to calibrate the system, by measuring the real temperature on the coverslip, at the single NCs position. The sample surface temperature is measured with a thermal camera (FLIR i5) placed above the microscope, which results in a real time temperature monitoring of the sample. When working with such kind of thermal imaging at the single NC level it is important to consider the target's thermal emissivity *ε* and reflectivity. A miscalibrated camera can generate thermal artifacts in the measurements as hotspots and misreadings. For the instrument used here the ambient temperature and the emissivity value of the coverslip can be configured in the camera. The room temperature was maintained between 293–295 K and the defined emissivity value was that of glass *ε*_glass_ = 0.95. For each measurement these values were checked out.

**Fig. 2:**
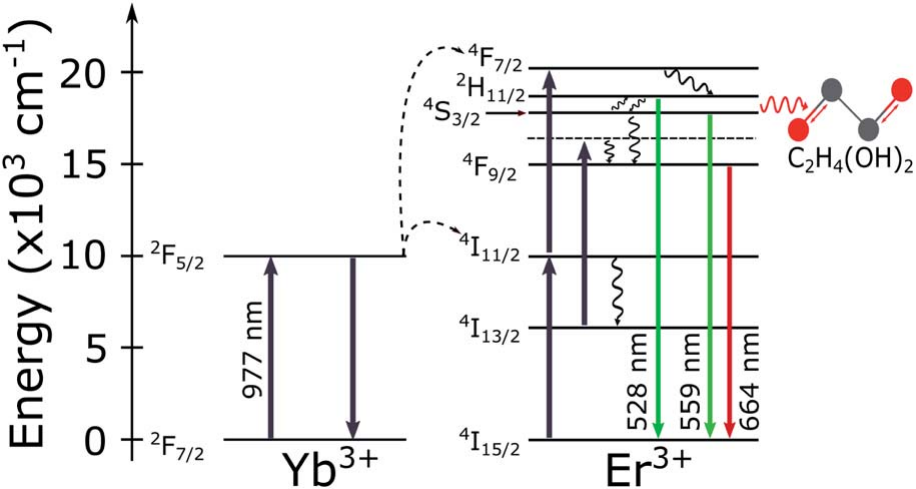
Energy level diagram for the Yb^3+^/Er^3+^ codoped systems. The solid arrows indicate radiative absorption/emission processes; curly arrows indicate multiphonon processes; the red curly arrow represents the phonon mediated energy transfer from the thermally coupled ^2^H_11/2_ and ^4^S_3/2_ states to the vibrational C–O (grey and red spheres, respectively) stretching modes of the ethylene glycol molecules.

The detected luminescence of the Yb^3+^/Er^3+^ codoped systems arises from well-established transition mechanisms^43^ as exemplified in Fig. 2. The photophysics that explains the excitation mechanisms responsible for the visible luminescence in such systems are the energy transfer upconversion (ETU), excited-state absorption (ESA) and ground state absorption (GSA). Out of the three excitation processes the ETU mechanism is the dominant one, since the absorption cross-section of the Yb^3+^ around 977 nm is much larger than for Er^3+^ ions.^10,44^ When the NCs are illuminated with 977 nm light, part of the Er^3+^ ions is promoted to the ^4^F_7/2_ excited state. Subsequent relaxation of the ^4^F_7/2_ state can populate the ^2^H_11/2_ (generating the emission band around 528 nm) and ^4^S_3/2_ (emission band around 559 nm) *via* fast nonradiative decay. The states ^2^H_11/2_ and ^4^S_3/2_ are sufficiently close in energy to become thermally coupled and their populations follow a Boltzmann distribution.^35,45^ However, the literature on thermometry using rare-earth-based materials often overlooks that important deviations from the Boltzmann population distribution are frequently present in experiments. These deviations from the Boltzmann distribution have been recently discussed and explained in detail.^36,46,47^ Nevertheless, for the purposes of the present work it is sufficient to use the LIR regarding the two thermally coupled green emission transitions to define the local temperature of the environment surrounding single NCs. Notice in particular that the processes shown in Fig. 2 suggest that the red emission should not be relevant in the present experiments. The thermally induced population redistribution between ^2^H_11/2_ and ^4^S_3/2_ should not be significantly affected by the Er^3+^ excitation pathway that involves the ^4^I_13/2_ state and the red emission at 664 nm as long as the ^4^F_9/2_ state is non-saturated.

As the main goal here is to investigate the same NCs in different surrounding media, it is necessary to identify regions with a convenient spatial distribution of individual NCs. A set of scanning luminescence images was taken, making possible to estimate which emitters are individual. Considering that the NCs have diameters between 70 and 150 nm (ref. 36) and the number of emitting ions is proportional to the NC volume, the luminescence intensity variation between the smaller and the larger NC sizes should be around 2×. The NCs suspension from the powder and the spin coating protocol used to make the samples guarantee that the vast majority of particles deposited on the coverslip happen as individual NCs, as confirmed by electron microscopy images.^36^ A selected 11 × 11 μm region with at least 8 NCs can be seen in the right panel of Fig. 1. To identify which particles are individual, it is considered the emission intensity of the faintest NC (particle 1, around 12 × 10^4^ counts per s) and twice this value as the maximum emission intensity of a single NC (around 25 × 10^4^ counts per s). The luminescence pattern shown in the Fig. 1 indicates that five particles are single NCs according to the analysis described above. While the 2× brightness criteria for larger NCs could also be associated with small particle dimers, the characterization below (see Section 3) indicates that all 5 particles follow similar trends in all parameters, which is an evidence that all NCs are individual. Those particles are indicated by white arrows in the Fig. 1 along with their respective identification numbers used throughout the manuscript.

**Fig. 3:**
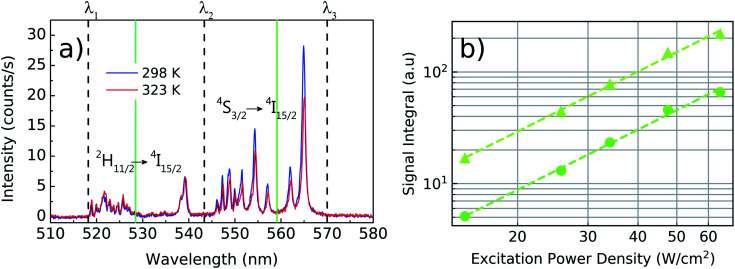
Luminescence spectra of the green emissions for a single Yb^3+^/Er^3+^:Y_2_O_3_ NC at 298 K (blue line) and 323 K (red line). On (a), the vertical dashed lines represents the wavelength intervals given by *λ*_1_ = 518 nm, *λ*_2_ = 543 nm and *λ*_3_ = 570 nm and the green solid lines represents the center wavelengths at 528 nm and 559 nm to indicate the set of transitions ^2^H_11/2_ → ^4^I_15/2_ and ^4^S_3/2_ → ^4^I_15/2_, respectively. Both spectra were obtained under an excitation power density of 64.2 W cm^−2^. On (b), the integrated emission intensity of the transitions ^2^H_11/2_ → ^4^I_15/2_ (*λ*_1_ to *λ*_2_, circles) and ^4^S_3/2_ → ^4^I_15/2_ (*λ*_2_ to *λ*_3_, triangles) is shown for various pump powers in a bilogarithmic scale. The slopes of the fitted curve are given by *n* = 1.8 ± 0.1 indicating that the transitions are pumped by a non-saturated two-photon process.

Once individual NCs have been detected, as seen in the Fig. 1, the excitation power dependence was investigated to check for saturation effects. This analysis was performed to ensure that the thermometric experiments will be carried out in a regime in which the nonradiative transitions dominate over the radiative ones, fact that is, unfortunately, also strongly overlooked in the literature related to nanothermometry with rare-earth doped nanoparticles. The emission spectrum of the green transitions and the saturation study can be seen in the Fig. 3a. As discussed in the ref. 36 the main peaks centered at 525 nm and 540 nm are correlated when the temperature varies. Also it can be seen that the multiple peaks around 550 nm and at 565 nm share identical variations with the temperature. Therefore, the emission bands in the spectral range from *λ*_1_ (518 nm) to *λ*_2_ (543 nm) are assigned as being related to the ^2^H_11/2_ → ^4^I_15/2_ transition, while the emission bands in the range from *λ*_2_ to *λ*_3_ (570 nm) are associated with the ^4^S_3/2_ → ^4^I_15/2_ transition. The two green vertical lines indicate the center wavelengths for both sets of transitions in Fig. 3 and are located at 528 nm and 559 nm. The luminescence intensity *I* in each transition is related with the pumping power *P* by *I* ∝ *P*^*n*^, where *n* is defined as the number of photons involved in the UC process. Here, *P* was measured after the objective lens and *n* is obtained by fitting the integrated values of *I*(*P*) in a bilogarithmic scale graph. The measurements result in a value of *n* = 1.8 ± 0.1 for pumping powers density ranging from 12.0 to 64.2 W cm^−2^ and the fit quality, with negligible deviations from the *n* = 1.8 trend, indicate that saturation effects can be neglected within this range. Despite being less intense due to the presence of thermal effects, the ^2^H_11/2_ → ^4^I_15/2_ transition becomes more prominent as the temperature of the system rises, allowing LIR measurements to be made even in a low intensity pumping regime. A working power density of 64.2 W cm^−2^ was defined to perform the thermometric measurements, with a total integration time of 60 s for each spectrum.

**Fig. 4:**
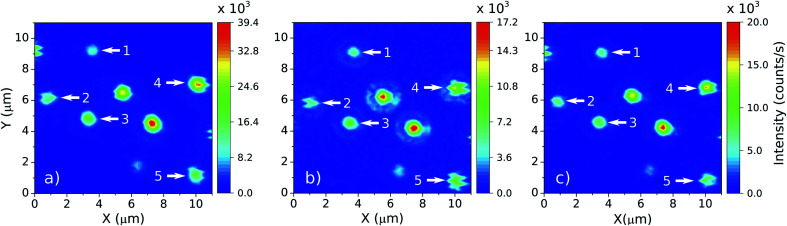
Emission patterns of the five selected NCs, indicated by the white arrows, in three media: (a) air (but after the evaporation of the water, from the thermometric LIR measurements with the NCs embedded in this medium), (b) water and (c) ethylene glycol. Notice that the NC constellation keeps its distribution in each medium, such that the data collected always refer to the same NCs.

In order to study the influence of the surrounding medium on the thermal response of individual NCs, it was established a protocol to deposit liquids in a controlled manner over the coverslip. This is particularly important, since the particles are not bonded to the surface and may be washed away when the liquids are deposited. With the particles still embedded in air, it is chosen a region with a convenient spatial distribution of five individual NCs. Then, a droplet of 100 μL of distilled water is carefully deposited over the sample with a micropipette. Before performing the thermal measurements, it is assured that the NC constellation has the same pattern observed in air (see Fig. 4b). Only then the LIR measurements of the NCs in water is started. Water is a troublesome liquid to work with because the droplet evaporates fast for temperatures higher than 305 K. It is thus necessary to refill the main droplet with additional 30 μL of water and wait for a thermal stabilization for every measurement at each new temperature value. If the main droplet dries, a “coffee sludge” effect may agglomerate the NCs and subsequently distinguishing them becomes impracticable. Once all the measurements are performed in water, the sample's temperature is reduced to the room temperature (around 292 K) and the water is left to evaporate naturally. This evaporation method minimizes the agglomeration effect and allows one to find the same spatial distribution of the NCs in air again, confirmed by subsequent luminescence scanning images (see Fig. 4a). After the water evaporates, the LIR measurements are performed in air. Once that the measurements in air are finished, a droplet of 100 μL of ethylene glycol is carefully deposited over the sample. Again the same NC luminescence pattern is retrieved (Fig. 4c) and the LIR thermal measurements are performed in this medium. Due to the slow evaporation of the ethylene glycol the sample does not need to be refilled.

## 3 Results and discussions

The two energy levels ^2^H_11/2_ and ^4^S_3/2_ are close enough in energy for the thermal coupling between these levels be relevant. Therefore, their occupation probability distribution obeys the Maxwell–Boltzmann statistics in such a way that the LIR (*R*) as function of the temperature is given by^45^1
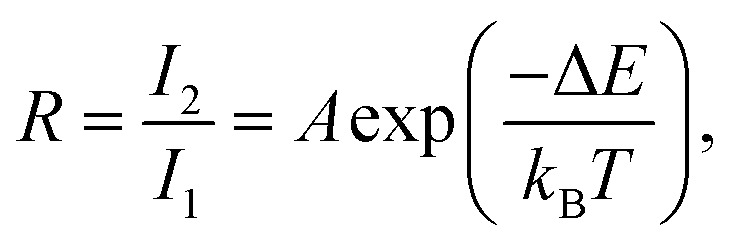
in which the two intensity bands are due to the transitions ^2^H_11/2_ (*I*_2_) and ^4^S_3/2_ (*I*_1_), *A* is a dimensionless constant related to their degeneracies (2*J* + 1) and spontaneous decay rates,^17^ Δ*E* is the effective energy gap between the levels, *T* is the absolute temperature of the system and *k*_B_ is the Boltzmann constant. The eqn (1) can be rewritten as2
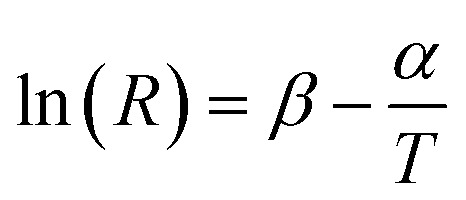
where *β* = ln(*A*) and *α* = Δ*E*/*k*_B_. Here *α* has dimension of temperature and gives information about the effective energy gap between the two coupled levels measured for the LIR while *β* is a dimensionless parameter. The common relation to quantify the efficiency of a ratiometric thermometer is given by the relative sensitivity, *S*_R_^11^3
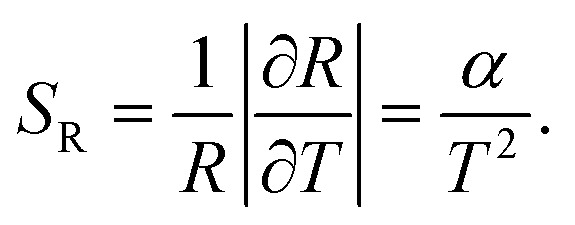


The relative sensitivity has dimension of % K^−1^ or K^−1^ and is useful to compare different thermometric systems.

For the three aforementioned media, the LIR measurements were performed in a temperature range from 293 K to 323 K in 10 steps according to the previously discussed temperature monitoring procedure. For each temperature value, the emission spectra were acquired one particle at a time, for the five selected particles. The LIR value for each particle was evaluated as the ratio between the numerical integration of the spectra in the bands [*λ*_1_, *λ*_2_] and [*λ*_2_, *λ*_3_].

**Fig. 5:**
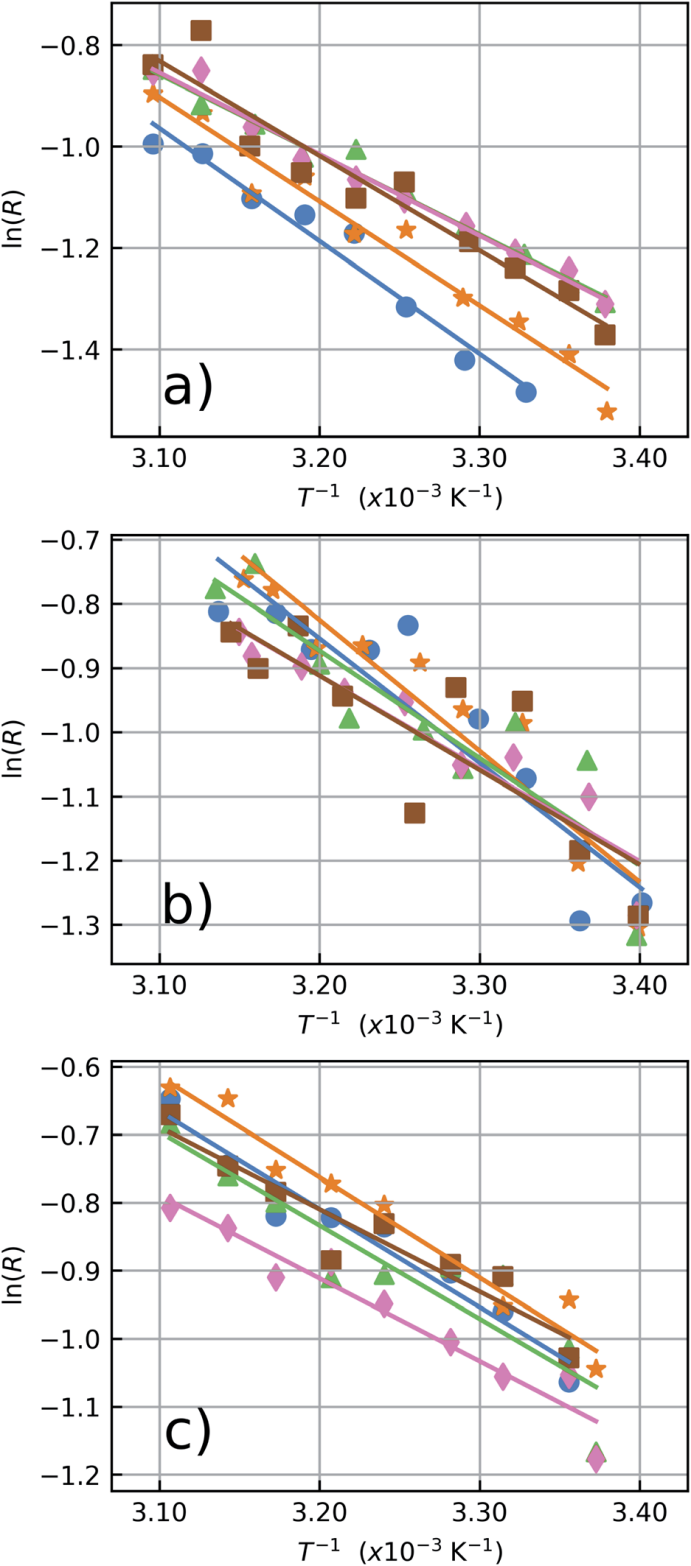
LIR thermometry measurements performed in (a) air, (b) water and (c) ethylene glycol as a function of inverse of the temperature. The solid lines are the linear fits used to determine *α* and *β* from the data corresponding to the particles 1 (blue circles), 2 (orange stars), 3 (green triangles), 4 (pink diamonds) and 5 (brown squares).

The LIR results for the five particles in air, water and ethylene glycol can be seen in Fig. 5 and the results are summarized in Table 1, in which the ensemble average values are also shown. The error values for the individual particles were extracted from the data fits and the ensemble error is taken as the standard deviation of the measured quantities. The individual measurements can be seen in the ESI Fig. S2–S4.†

**Table 1:** Measured parameters for the five selected particles extracted from linear fittings to experimental data by using eqn (2). Here Δ*E* = *k*_B_*α*. The *S*_R_ and *δT* values are given at 310 K

NP	Air				Water				Ethylene glycol			
	Δ*E* (cm^−1^)	*β*	*S*_R_ (% K^−1^)	*δT*	Δ*E* (cm^−1^)	*β*	*S*_R_ (% K^−1^)	*δT*	Δ*E* (cm^−1^)	*β*	*S*_R_ (% K^−1^)	*δT*
1	1550 ± 120	5.9 ± 0.6	2.3 ± 0.2	0.6	1350 ± 220	5.3 ± 1.1	2.0 ± 0.3	1.4	1001 ± 97	3.8 ± 0.5	1.5 ± 0.2	0.8
2	1427 ± 95	5.5 ± 0.4	2.1 ± 0.1	0.6	1420 ± 180	5.7 ± 0.8	2.1 ± 0.3	1.1	1028 ± 77	4.0 ± 0.4	1.6 ± 0.1	0.6
3	1095 ± 48	4.0 ± 0.2	1.6 ± 0.1	0.4	1160 ± 220	4.5 ± 1.0	1.7 ± 0.3	1.7	950 ± 160	3.6 ± 0.7	1.4 ± 0.2	1.4
4	1116 ± 52	4.1 ± 0.2	1.7 ± 0.1	0.4	1000 ± 130	3.7 ± 0.6	1.5 ± 0.2	1.2	846 ± 86	3.0 ± 0.4	1.3 ± 0.1	0.9
5	1300 ± 130	5.0 ± 0.6	1.9 ± 0.2	0.9	1020 ± 260	3.8 ± 1.2	1.6 ± 0.4	2.3	840 ± 120	3.1 ± 0.5	1.3 ± 0.2	1.1
Ensemble average	1300 ± 150	4.9 ± 0.7	2.1 ± 0.2	2.5	1190 ± 156	4.6 ± 0.7	1.9 ± 0.2	4.3	934 ± 72	3.5 ± 0.4	1.5 ± 0.1	2.7

The spectroscopic energy gap between the two sets of energy levels [*λ*_1_, *λ*_2_] and [*λ*_2_, *λ*_3_], when the center wavelengths of 528 nm and 559 nm are considered, results in Δ*E*_spec_ = 1050 cm^−1^. In air, it can be noticed that the measured effective gap energies, Δ*E* = *k*_B_*α*, are larger than Δ*E*_spec_. For instance, the result obtained for the particle 1 in air was Δ*E* = 1550 ± 120 cm^−1^. Besides, the measured values presented here are greater than the Δ*E* values measured by Galvão *et al.*^36^ for the same kind of particles. The different basic excitation conditions used in both works (the two orders of magnitude difference in pumping power and the very different bandwidths of the excitation lasers used, which influences the number of ion classes excited within the inhomogeneous broadened emission lines) can explain these variations in sensitivity. This further corroborates the ansatz sustained in previous works^36,46^ that the thermometer accuracy and performance strongly depends on the way the thermally coupled energy levels are populated, and that not taking this into account may lead to inaccurate temperature measurements. In addition, according to Marciniak and collaborators^25^ the smaller Δ*E* at higher pump powers can be explained through heating induced by the absorption of NIR photons. Their work reports differences on the sensitivity by an order of magnitude from low to high excitation regimes with values of *S*_R_ = 2.66% K^−1^ and *S*_R_ = 0.28% K^−1^, respectively. This argument is reinforced by the fact that nonradiative transitions for low lying energy levels increase as the energy difference between them decreases.^25,48^ Pickel *et al.* reported “apparent” self-heating effects induced by high excitation power densities (above 10^4^ W cm^−2^),^49^ since the temperature artifacts are not a result of poor heat dissipation from the NC to its surroundings, but instead, is explained by a non-Boltzmann distortion of the ^2^H_11/2_ state population due to multiphonon decay processes from high lying levels. These effects could significantly change the LIR values and generate temperature artifacts which must be considered when working with individual NCs. Thus, to avoid this kind of temperature misreading due to high excitation power densities, the LIR measurements in the present work were performed at a constant excitation power density of 64.2 W cm^−2^ which is several orders of magnitude below the necessary for the manifestation of “apparent” self-heating effects.

The opposite effect is observed at low pumping powers. In this regime, the effects populating the high lying state due to thermalization are dominant. Higher sensitivities can be achieved at low pumping powers but it also leads to higher integration times along with a poorer signal-to-noise relation, which increases the measurement uncertainties. The measured ensemble average Δ*E* values in air and water agree with each other within a standard deviation and also agree with previous reports in the literature.^20,50–54^ Concerning the relative sensitivity, it were measured values up to *S*_R_ = 2.3% K^−1^ at 310 K (see Table 1). The relative sensitivity values for Yb^3+^/Er^3+^ codoped oxide matrices are found in the literature within a range from 0.3% K^−1^ to 2.8% K^−1^ at ≈300 K.^25^ Our measurements in water and ethylene glycol have poorer signal to noise ratios in this pumping regime. Compare Fig. 5a–c. This occurs because the luminescence signal drops to approximately half of its value in air when the liquids are deposited over the sample due to increase in the refractive index surrounding the NCs. Which enhances the probability of photon emission towards the medium with the higher refractive index.^55^ The similar thermal behaviour of the NCs in both environments, water and air, reinforces the assumption that these NCs are suited for nanothermometric measurements in aqueous systems such as biological media (around 70% of water) as shown in other works.^34,56^ The Δ*E* measurements for the NCs in ethylene glycol are consistently smaller than those in air and water (see Fig. 5c). This reduction in Δ*E* can be explained considering the influence of the strong ethylene glycol vibrational modes^57,58^ around 1044 cm^−1^. The C–O bond stretching vibrational mode has a strong response and is almost resonant with the energy gap between the two coupled states (Δ*E*_spec_ = 1050 cm^−1^), leading to the nonradiative depopulation of the higher lying^34^ state ^2^H_11/2_ which changes the population distribution of the thermally coupled states. This effect is represented by the wavy red arrow in the Fig. 2. It can be noticed that the ensemble average (see Table 1) present greater error values than the individual ones. In a case where an ensemble measurement is performed it does not consider the thermal response of each individual NC. Depending on the synthesis procedure, the NCs can possess high dispersion over several parameters as dopant concentrations, shape, size, surface defects, *etc.*^10^ As will be shown later, intensity luminescence measurements can relate the size of an individual NCs to the distinct measured values for Δ*E*. Therefore, the error bars for individual NCs do not contain variations in the thermal response due to dimensional inhomogeneity which is present in the ensemble average.

At an individual level, the NCs have distinct thermal responses among each other (Table 1). This results can be related to variations in the NCs' size^33,59^ caused by surface quenching sites^60^ and micro-strain effects.^61^ An estimate based on the intrinsic stress caused by micro-strain in Y_2_O_3_ micro^62^ and nanoparticles^63^ results in stress values on the order of 1.0 GPa on the NCs. While we are unaware of specific studies relating strain and emissions on our system, there are reports on other doped yttrium-based crystal matrices. Recent studies on codoped YF_3_:Yb^3+^/Er^3+^ microparticles^64^ and YVO_4_:Yb^3+^/Er^3+^ NCs^65^ under high pressure reported no significant changes on the LIR of the green emission bands with increasing stress (up to 10.0 GPa). Since our micro-strain estimate is one order of magnitude smaller than those reported for significant effects on the LIR of the green emission, we believe that the micro-strain effects does not play a primary role in our LIR signal and the associated thermometric parameters. In the other hand, size dependent-quenching effects on the LIR of NCs were already reported.^33,56,59,66^ For instance, nanometric particles composed by^59^ LiLaP_4_O_12_:Cr^3+^/Nd^3+^ and^33^ Yb^3+^/Er^3+^ complexes present changes in the sensitivity from 1% K^−1^ to 5% K^−1^ for the first codoped nanocrystals and from 1.1% K^−1^ to 2.1% K^−1^ for the last one. The physical mechanisms responsible for this dependence rely on the optical quenching caused by the energy adsorbed by ions located at the surface of the NC and its fast nonradiative decaying rates.^33^ The participation of the surface ions relative to those located within the NC is inversely proportional to the radii of the NC.^33^ These surface sites have different spectroscopic features when compared to those “bulk” ions such as the presence of absorption bands and lack of emission for some selected sites.^67,68^ Therefore, it is assumed that beyond the already known decay processes in small NCs as nonradiative phonon assisted quenching and cross relaxations processes, the surface-related nonradiative quenching can occur. Further evidence for the relevance of these surface-related mechanisms in our nanothermometers is given below.

The values for *S*_R_ of the more (NC1) and less (NC3) sensitive nanothermometers in air are given by *S*_R(1)_ = 2.3 ± 0.2% K^−1^ and *S*_R(3)_ = 1.6 ± 0.1% K^−1^, respectively. In water, these values are given by *S*_R(2)_ = 2.1 ± 0.3% K^−1^ and *S*_R(4)_ = 1.5 ± 0.2% K^−1^ being equivalent to those in air within the experimental uncertainty. In water the most sensitive thermometers were the NCs 1 and 2 the emitters with the less intense detected luminescence. The larger errors measured in the presence of liquids are due to the reduction of the signal-to-noise ratio caused by the emission quenching in both environments which leads to a decrease in the detected luminescence intensity from NCs approximately given by *I*_water_/*I*_air_ = 0.51 and *I*_ethylene glycol_/*I*_air_ = 0.46.

In ethylene glycol, the maximum and minimum sensitivity values are given by *S*_R(1)_ = 1.6 ± 0.2% K^−1^ and *S*_R(5)_ = 1.3 ± 0.1% K^−1^, respectively. These values are very similar for all five NCs in this medium considering their error bars. The small variation of sensitivity values in ethylene glycol among all studied NCs should be associated with the surface effects associated with the molecular vibrational modes of this solvent, which were not present in the two other studied media (air and water).

The thermal resolution *δT* (see the ESI† for our approach to determine it from measurements over individual particles), *i.e.*, the smallest change in temperature the sensor is able to detect confidently^1^ for the five NCs at 310 K are reported in Table 1. The smallest values in air were found for the NC3 and NC4 with *δT* ∼ 0.4 K. For the water and ethylene glycol environments these values are given by *δT* ∼ 1.1 K and ∼0.6 K, respectively, for the NC2. The measured values for the thermal resolution depends not only on the *S*_R_ but also on the standard deviation of Δ*E* and *β* extracted from the LIR which are higher for the two liquid environments (due to the poorer signal-to-noise ratio) leading to high *δT* values. Here, the brighter emitters are the less sensitive ones and *vice versa*. Therefore, a balance between sensitivity and the NC emission intensity should be considered in order to optimize the thermal resolution of the optical nanothermometers. The definition of a thermal resolution from ensemble data, which considers the standard deviation of the averaged parameters,^36,46^ results in less precise thermal measurements with *δT* as high as 4.3 K. This can be explained by the differences on the measured Δ*E* and *β* parameters for the five NCs. The typical values for ensembles of UC lanthanide-doped NCs ranges from ^14^*δT* ∼ 1.0 K to 0.1 K in concordance with the measured values for the single NCs reported here.

For the ensemble averages shown in the Table 1, the *δT* values are obtained from the standard deviations of the average LIR values (see ESI†) for the five NCs at 310 K and the *S*_R_ is taken as the average over the relative sensitivity of the five NCs.

**Fig. 6:**
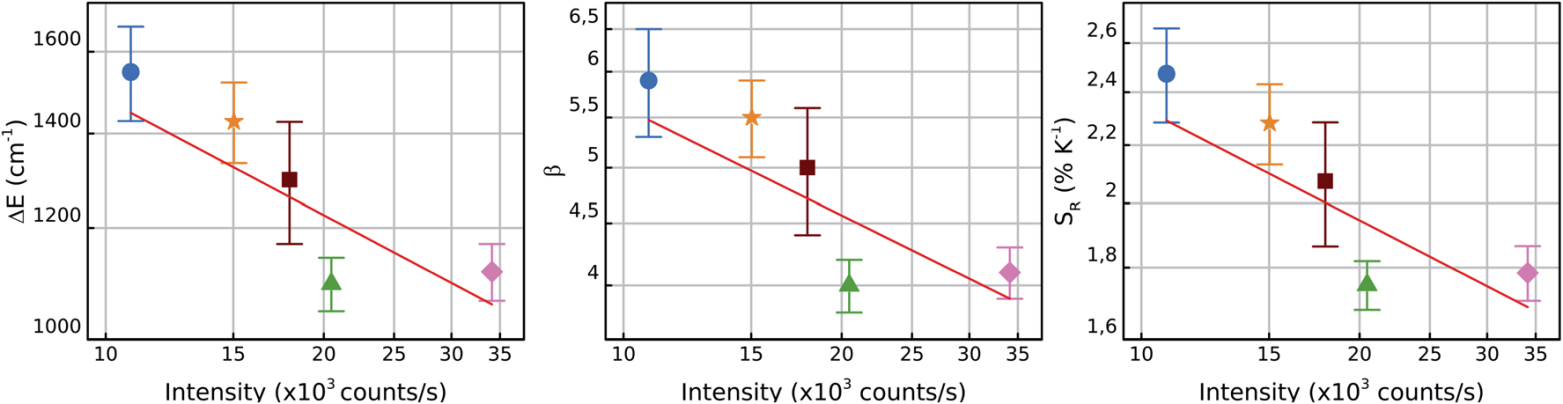
From left to right: bilogarithmic plots for Δ*E*, *β* and *S*_R_ for particles 1 (blue circles), 2 (orange stars), 3 (green triangles), 4 (pink diamonds) and 5 (brown squares) in air as function of the total NC luminescence intensity under 6 × 10^3^ W cm^−2^ of excitation power density at the NC. The red lines have a slope of ≈−0.3 for all three parameters. Since *I* ∝ volume ∝ *r*^3^, the slope of −0.3 indicates that these parameters vary according to 1/*r*, which is proportional to the surface/volume ratio.

In order to investigate the manifestation of surface effects as a function NC size, Fig. 6 shows the measured values of Δ*E*, *β* and *S*_R_ in air for each particle as function of the luminescence intensity *I*. Since the number of fluorescent ions grow with the NC volume, it is expected that *I* ∝ *r*^3^, where *r* is the NC radius. Therefore, the luminescence intensity is a proxy for an *in situ* NC radius measurement. In a bilogarithmic scale, the results show a linear dependence of all measured parameters on the luminescence intensity with a slope −0.3. This behavior is attributed to surface effects which depend on the number of surface sites relative to the “bulk” ions, since surface/volume ∝ 1/*r* ∝ *I*^−1/3^. Therefore, the higher surface-to-volume ratio, the higher will be the measured Δ*E* between the thermally coupled levels and consequently the *S*_R_ values.

The *β* parameter also varies with *I* and is related to the radiative lifetimes *γ*^rad^_*i*_ and the wavelengths *λ*_*i*_ of the two thermally coupled levels in the form^46,56,66,69^4
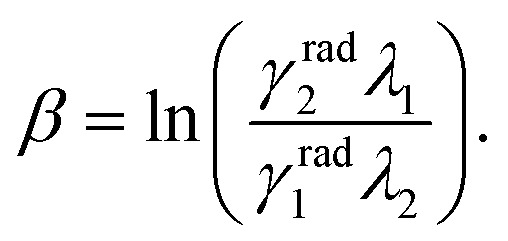


One possible explanation for this result is the dependence of the ^4^S_3/2_ mean decay rate in this kind of systems with the size of the NCs as shown in the ref. 60 and 70. These works show that the ^4^S_3/2_ mean lifetimes become shorter with the reduction of the NCs size and this could be justified by the presence of surface quenching effects, thus the mean decay rates present linear dependence with the surface/volume ratio.^60^ However, more investigations must be done in this matter to better understand this phenomenon. Hence, the individual characteristics of these emitters are particularly important when working in the individual level and brings up some individual effects which are not considered so far in ensemble measurements.

The simple relation between the parameters presented in the Fig. 6 with the intensity of the luminescence emitted by the NCs indicates that, in principle, one could perform the thermal calibration for one NC and interpolate the Δ*E*, *β* and *S*_R_ values with the information about its luminescence intensity given by the general dependence *I*^−1/3^. This result could reduce significantly the amount of work needed for the characterization of several individual NCs on a sample. To reinforce this argument, further investigations with individual nanothermometers are needed in order to better understand the physical mechanisms that lead to results other than those discussed here.

The results discussed above have direct consequences for several applications. For instance, consider a system where a single particle can be manipulated with nanometric spatial precision,^71^ say with the use of an optical tweezer,^72,73^ AFM manipulation^74^ or SNOM^75,76^ techniques. To perform high spatial resolution nanothermometry with the highest sensitivity as possible, the individual characteristics of the emitters must to be taken into account. Then, the NC with the best signal to noise ratio compromise could be chosen as the nanoscopic thermometer and then this sensor can be calibrated for target environment. If the research aim is to measure temperatures inside biological environments (about 70% of water), the thermal response should be similar to those nanothermometers calibrated in air, as shown here and in the ref. 13, 34 and 56. For industrial applications in non-biological environments, however, the physical aspects of the medium needs to be considered and the nanothermometer must be calibrated for that specific environment. The results discussed herein also applies in systems containing several NCs. Suppose a sample which has individual NCs, sufficiently spaced to be resolved in the imaging system, as for example in a wide field image. Given the particle brightness scaling of the thermometric parameters, it is possible to calibrate the thermal response of a few NCs and automatically extend that calibration to the entire illuminated region. This could be particularly useful to measure hot-spots in micro-/nano-electronic failure diagnosis assessments.

## 4 Conclusions

In this work, the same five luminescent nanothermometers composed by individual Y_2_O_3_:Yb^3+^/Er^3+^ NCs have been characterized in a temperature range from 293 K to 323 K. The investigations considered the influence of the surrounding medium (air, water and ethylene glycol) on the NCs thermometric sensitivity obtained through LIR measurements performed under CW laser excitation at 977 nm. The LIR results show that the relative sensitivity presented by the NCs differ from each other in the three investigated media with relative sensitivities obtained in air, water and ethylene glycol given by 2.5 ± 0.2% K^−1^, 2.2 ± 0.4% K^−1^ and 1.6 ± 0.2% K^−1^, respectively. The best thermal resolution obtained for the individual NCs were 0.4 K, 1.1 K and 0.6 K in air, water and ethylene glycol, respectively. The thermal resolution depends on the relative errors of the effective energy gap Δ*E* and the dimensionless parameter *β*, which are systematically higher in water. In air, the five investigated NCs present a discrepancy in the maximum relative sensitivity of ≈29%. This discrepancy reduces to ≈9% in ethylene glycol. The thermometer's sensitivity in ethylene glycol is reduced relative to the air and water. This behavior can be explained through the enhancement of the nonradiative decay of the ^2^H_11/2_ state due to the coupling to vibrational stretching modes of the C–O bonds of the ethylene glycol molecules. These results indicate that in specific situations the calibration of lanthanide-ion based luminescent nanothermometers can be strongly affected by its interaction with the surrounding medium. Besides that, the presence of a liquid environment around the particle assures the thermal equilibrium as well as simulates the conditions found in biological systems.

It was also noticed that the higher sensitivities were observed in faintest particles. Since the particle brightness must be proportional to the particle volume, this indicates that smaller particles have an enhanced sensitivity. This implies that the studied system exhibits a trade-off between sensitivity and signal to noise ratio. It was also found that the thermometric parameters used here, Δ*E*, *β* and *S*_R_, all vary with the mean NC brightness *I* as *I*^−0.3^. The proportionality between *I* and the volume suggests that the exponent −0.3 is directly related to the surface/volume ratio. Therefore, the different sensitivities should be related to surface-related non-radiative quenching effects, as surface defects or coupling to nearby molecules.

## Conflicts of interest

There are no conflicts to declare.

## Supplementary Material

NA-003-D1NA00466B-s001
