# Correction: Influence of the surrounding medium on the luminescence-based thermometric properties of single Yb^3+^/Er^3+^ codoped yttria nanocrystals

**DOI:** 10.1039/d1na90104d

**Published:** 2021-11-17

**Authors:** Jefferson Augusto Oliveira Galindo, Allison Rodrigo Pessoa, Anderson Monteiro Amaral, Luiz F. dos Santos, Rogéria R. Gonçalves, Leonardo de Souza Menezes

**Affiliations:** Department of Physics, Universidade Federal de Pernambuco – UFPE Recife PE 50670-901 Brazil anderson.amaral@ufpe.br +55-81-2126-7640; Laboratório de Materiais Luminescentes Micro e Nanoestruturados – Mater Lumen, Departamento de Química, FFCLRP, Universidade de São Paulo Ribeirão Preto SP Brazil

## Abstract

Correction for ‘Influence of the surrounding medium on the luminescence-based thermometric properties of single Yb^3+^/Er^3+^ codoped yttria nanocrystals’ by Jefferson Augusto Oliveira Galindo *et al.*, *Nanoscale Adv.*, 2021, **3**, 6231–6241. DOI: 10.1039/D1NA00466B.

The authors regret the omission of two authors, Prof. Rogéria R. Gonçalves and Luiz F. dos Santos, from the original manuscript. The corrected author list is as shown above.

In addition the authors acknowledge the financial support from the Brazilian science funding agencies Coordenação de Aperfeiçoamento Pessoal de Nìvel Superior (CAPES), Conselho Nacional de Desenvolvimento Científico e Tecnológico – (CNPq) (grant number: 303110/2019-8 and 142199/2020-6 for the scholarship), Ministério da Saúde (PRONON-SIPAR Project #25000.077093/2015-86), Fundação de Amparo à Pesquisa do Estado de São Paulo (FAPESP, grant number: 2020/05319-9), Fundação de Amparo à Ciência e Tecnologia do Estado de Pernambuco (FACEPE) and the National Photonics Institute – INFo/CNPq.

The Royal Society of Chemistry apologises for these errors and any consequent inconvenience to authors and readers.

## Supplementary Material

