# Supplementary material for: Influence of the surrounding medium on the luminescence-based thermometric properties of single Yb3+/Er3+ codoped yttria nanocrystals
Source: Nanoscale Adv. 2021 Sep 16;3(21):6231–41. doi: 10.1039/d1na00466b (PMC9417197; doi:10.1039/d1na00466b)
Supplement: NA-003-D1NA00466B-s001 [file NA-003-D1NA00466B-s001.pdf]

Electronic Supplementary Material (ESI) for Nanoscale Advances.
This journal is © The Royal Society of Chemistry 2021

Supplementary Information - Influence of the surrounding medium on the luminescence-based thermometric properties of single Yb^{3+}/Er^{3+} codoped yttria nanocrystals

J. A. O. Galindo,[†] A. R. Pessoa,[†] A. M. Amaral,[*,†] and L. de S. Menezes[†‡]

†*Department of Physics, Universidade Federal de Pernambuco (UFPE), 50670-901, Recife-PE, Brazil*

‡*Present address: Chair in Hybrid Nanosystems, Nanoinstitut München, Fakultät für Physik, Ludwig-Maximilians-Universität München, 80539 München, Germany*

E-mail: anderson.amaral@ufpe.br

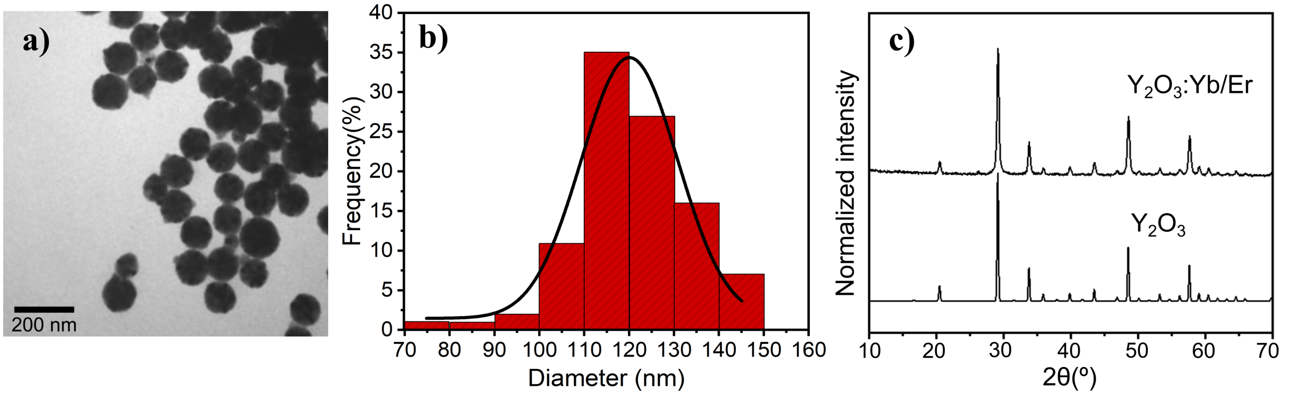

Figure S1: a) Transmission Electron Microscopy (TEM) image of multiple Y_2O_3:Yb^{3+}/Er^{3+} NCs b) Size distribution of the Yb^{3+}/Er^{3+} codoped NCs. The particles can be found in sizes ranging from ≈ 70 nm to ≈ 150 nm with average value of 120 ± 20 nm. c) Diffractogram of the codoped Y_2O_3:Yb^{3+}/Er^{3+} and pristine Y_2O_3 confirming the body centered cubic phase structure of the NCs.

SI1. Morphological characterization of Y_2O_3 NCs

The sample preparation protocol leading to sparse single NCs on a glass coverslip has been already described by Galvão *et al.*, in reference.[1] Their work contains SEM and TEM images of single NCs before and after the spin coating technique used for preparing the samples (see subsection 2.1 and Figure 1c) of reference[1] and Figure 4a) of reference[2] for another sample - Nd^{3+} doped ytrria NCs following the same sample preparation protocol). Their results show that the spin coating technique herein used leads to a majority of single particle sites spread all over the glass coverslip surface.

SI2. Individual LIR measurements for the five selected Y_2O_3 codoped NCs in air, water and ethylene glycol

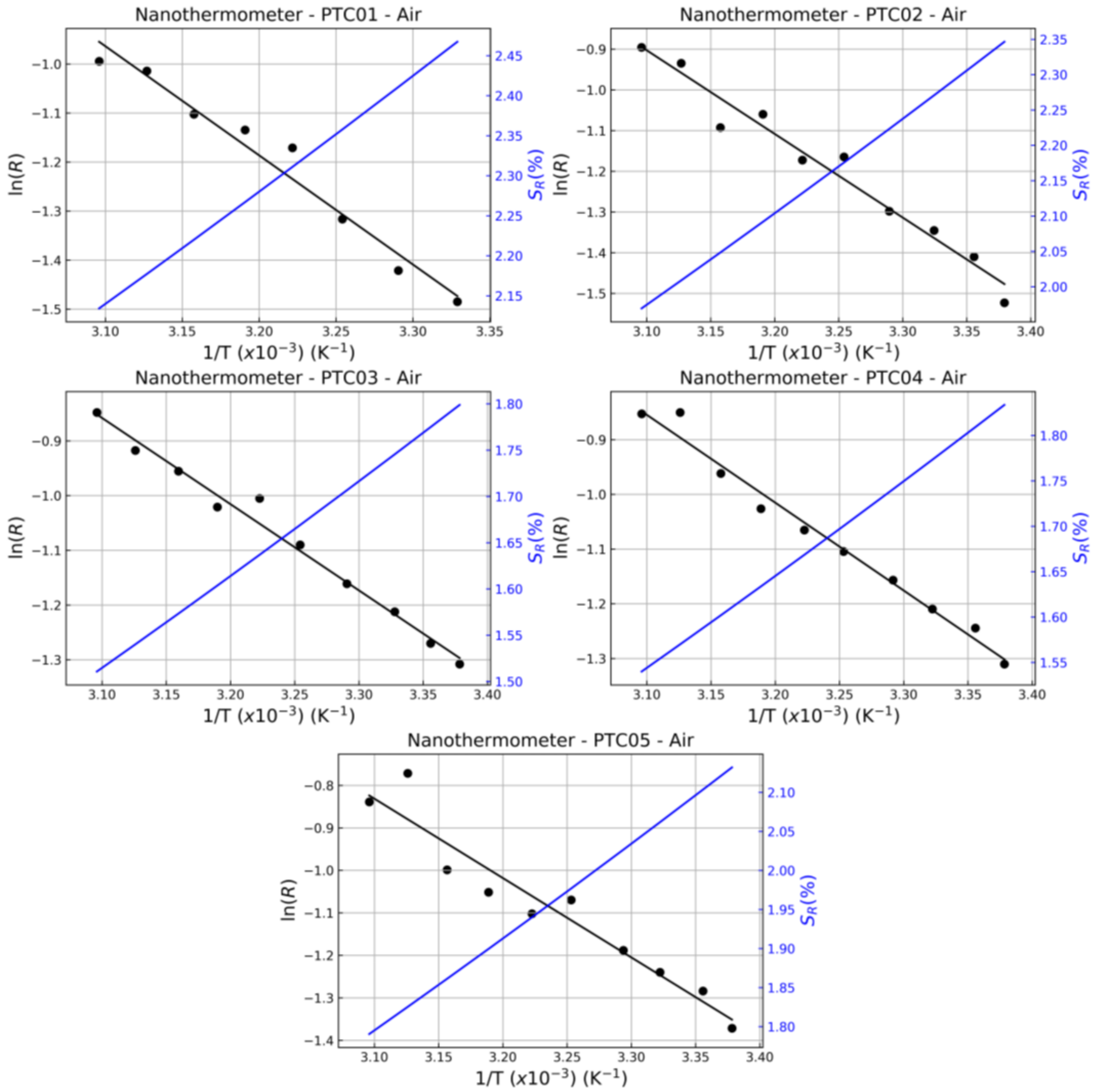

Figure S2: Individual measurements for the five NCs in air. The black (blue) line is the LIR (S_R) fit (calculated values). The measured values for β, ΔE and S_R are given in Table 1.

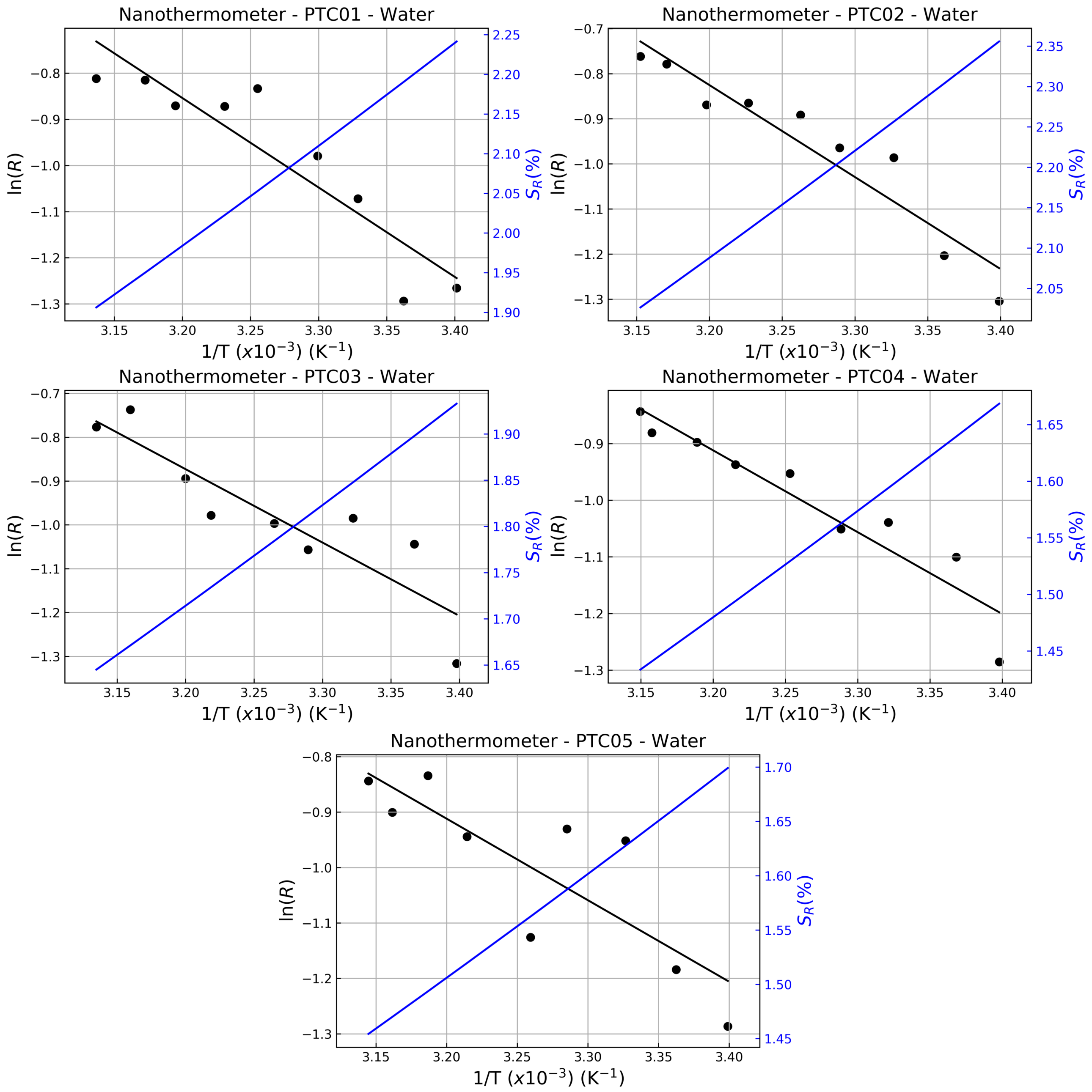

Figure S3: Individual measurements for the five NCs in water. The black (blue) line is the LIR (S_R) fit (calculated values). The measured values for β, ΔE and S_R are given in Table 1.

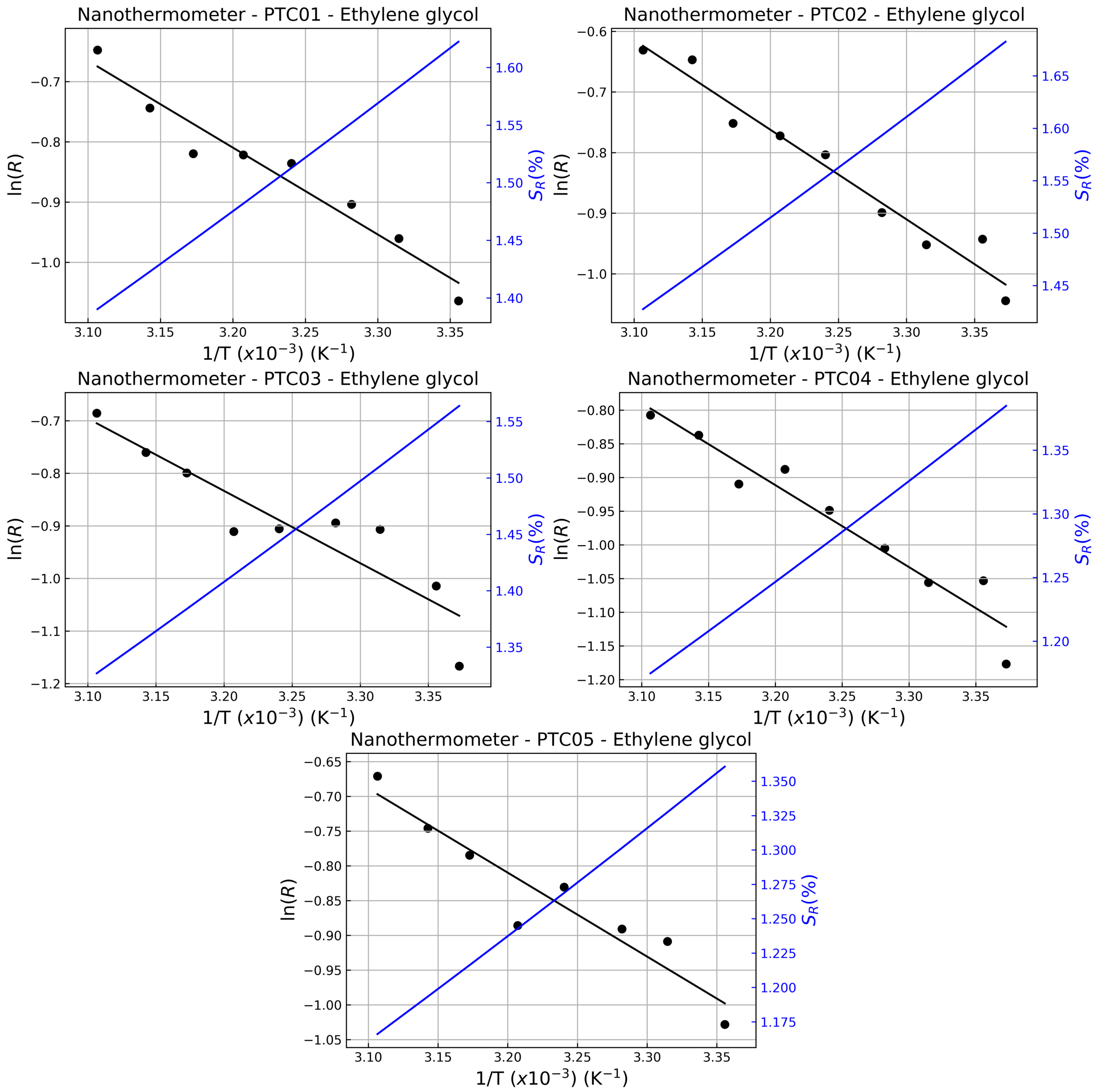

Figure S4: Individual measurements for the five NCs in ethylene glycol. The black (blue) line is the LIR (S_R) fit (calculated values). The measured values for β, ΔE and S_R are given in Table 1.

SI3. Thermal resolution of individual nanocrystals (NCs)

The thermal resolution of a thermometer (δT) is defined as the minimum temperature change that the system is able to measure confidently,[3] thus it is a function of the LIR (R). One can expand δT in Taylor's Series and truncate to the first non-vanishing term, which results in

$$\delta T = \frac{1}{S_R}\frac{\delta R}{R}$$

According to Brites *et. al.*,[3] the uncertainty in the determination of the LIR (δR) can be calculated either by measuring a set of LIR values in the same experimental condition, making a histogram and calculating the standard deviation; or by propagating the uncertainties from the signal-to-noise ratio of the detection system. Even though the above-mentioned procedures are well-established, they are not suitable to individual calibration in our system. A different approach which can also be used to gain more physical intuition about the system is discussed below.

As reported in the main text, the calibration of the thermometer is made by fitting the LIR vs. Temperature data and obtaining two parameters: α, and β (equation 2 in the main manuscript). The fitting is performed by standard linear regression, in which it is also possible to calculate the variance-covariance matrix, defined by[4]

$$\Sigma = \begin{bmatrix} \sigma^2(\beta) & \mathrm{Cov}(\beta,\alpha) \\ \mathrm{Cov}(\alpha,\beta) & \sigma^2(\alpha) \end{bmatrix}$$

where

$$\sigma^{2}(\gamma) = E\left\{[\gamma - E(\gamma)]^{2}\right\}$$

is the variance of the variable $\gamma = \alpha$ or β, where $E[\gamma]$ is its the expected value, and

$$\text{Cov}(\alpha, \beta) = \text{Cov}(\beta, \alpha) = E\{[\beta - E(\beta)][\alpha - E(\alpha)]\}$$

is the covariance of the two variables α and β.

If the two variables are independent random variables, the covariance between them must vanish, and the variance-covariance matrix becomes diagonal. Thus each element of the diagonal completely characterizes the statistical properties of its corresponding parameter. In the opposite situation, where the variables are not independent, the Σ matrix is not diagonal, but still symmetric.

In the present work, however, by performing the linear regression via the Least Mean Square algorithm, it was observed that the covariance matrices for all thermometers fittings are not diagonal, which means that α and β are correlated. The physical meaning for this correlation relies on the choice of the Boltzmann Law to model the photophysical dynamics of the system. As discussed in a very recent work,[5] the authors used the same codoped system in a $NaYF_4$ matrix and showed that the dynamics of non-radiative absorption and decay of the Er^{3+} ions leads to a deviation of the Intensity Ratio from the Boltzmann law, for the same thermally coupled levels used in this work.

Naturally, the β parameter depends on the radiative decay rates (equation 5 in the main manuscript), but the authors showed that the ΔE parameter evaluated by the Boltzmann Law is actually not the spectroscopic value, but an apparent value that depends also on the radiative decay rates. Therefore, α and β must be correlated in experiments. An important consequence is that the error propagation to obtain derived quantities as the thermal resolution must consider the off-diagonal terms in Σ.

In order to determine δR, it is possible to set that, for a fixed temperature T_0, $X_0 = 1/T_0$ and R as $R(\alpha, \beta)$. Thus, the δR can be written in matrix form[4]

$$(\delta R)^2 = \begin{bmatrix} 1 & X_0 \end{bmatrix} \cdot \begin{bmatrix} \sigma^2(\beta) & \text{Cov}(\beta, \alpha) \\ \text{Cov}(\alpha, \beta) & \sigma^2(\beta) \end{bmatrix} \cdot \begin{bmatrix} 1 \\ X_0 \end{bmatrix}$$

The subsequent calculations of all related quantities presented in Table 1 follows from standard error propagation.

SI4. "Ensemble" averages and thermal resolution

The "ensemble" values presented in Table 1 for ΔE , β and S_R were extracted from the mean of the values for the five selected NCs. The error bars for those values were defined as the standard deviation from the mean for each parameter.

The thermal resolution for the ensemble ($\delta T^{(e)}$) was obtained according to the references ([1,5]), being applicable for systems with multiple micro/nano-thermometers at a fixed temperature (T_0) and depends on the standard deviation (σ) of the LIR values for the studied NCs through

$$\delta T^{(e)} = \frac{1}{S_R^{(e)}} \frac{\sigma}{R^{(e)}}$$

where the superscript (e) holds for the ensemble mean values for R and S_R . The values for $\delta T^{(e)}$ presented in Table 1 are higher than the obtained thermal resolution for the individual NCs because the dispersion of the LIR values for the set of nanothermomethers leads to a high standard deviation.

References

(1) Galvão, R.; dos Santos, L. F.; Lima, K. de O.; Gonçalves, R. R.; Menezes, L. de S. Single Er^{3+}/Yb^{3+}-Codoped Yttria Nanocrystals for Temperature Sensing: Experimental Characterization and Theoretical Modeling. J. Phys. Chem. C **2021**, 125, 14807–14817.

(2) Camargo, K. C.; Pereira, R. R.; dos Santos, L. F.; de Oliveira, S. R.; Gonçalves, R. R.; Menezes, L. de S. Continuous wave near-infrared phonon-assisted upconversion in single Nd^{3+}-doped yttria nanoparticles. J. Lumin. **2017**, 192, 963–968.

(3) Brites, C. D. S.; Millán, A.; Carlos, L. D. In Including Actinides; Jean-Claude, B., Vitalij K., P., Eds.; Elsevier, 2016; Vol. 49; pp 339–427.

(4) John, R. C. St.. Applied linear regression models. 1983.

(5) Gonçalves, I. M.; Pessoa, A. R.; Hazra, C.; Correales, Y. S.; Ribeiro, S. J. L.; Menezes, L. de S. Phonon-assisted NIR-to-visible upconversion in single β-$NaYF_4$ microcrystals codoped with Er^{3+} and Yb^{3+} for microthermometry applications: Experiment and theory. J. Lumin. **2021**, 231, 117801.
